# Examining the psychometric properties of the Turkish version of the proactive coping scale in nursing students: A methodological study

**DOI:** 10.1186/s12912-024-02150-1

**Published:** 2024-07-15

**Authors:** Esra Özbudak Arıca

**Affiliations:** Faculty of Health Sciences, Yozgat Bozok University, Yozgat, Turkey

**Keywords:** Nursing students, PROACTIVE coping, Translation, Turkish, Validity and reliability

## Abstract

**Aim:**

The aim of this study was to use the “PROACTIVE Coping Scale” to adapt the scale to Turkish culture, to determine its validity and reliability in a sample of undergraduate nursing students, and to evaluate the proactive coping levels of nursing students.

**Background:**

Proactive coping skills are very important for nursing students to cope effectively with various stressors that they may encounter both in their academic lives and in their future professional lives. There are no valid and reliable instruments for measuring the proactive coping levels of nursing students in Turkey.

**Methods:**

The present study is a descriptive and methodological study. Research data were collected between 01.12.2023 and 01.01.2024 via face-to-face interviews. The study was completed by 272 nursing students who voluntarily agreed to participate in the study. In the analysis of the data, number/percentage, exploratory and confirmatory factor analysis, and Cronbach’s Alpha reliability coefficient methods were used.

**Results:**

The scale structure was confirmed with 19 items and 4 factors. The Cronbach’s alpha reliability coefficient of the PROACTIVE Coping Scale was found to be 0.816. The scale explains 67.17% of the total variance, and item correlation values vary between 0.263 and 0.650.

**Conclusions:**

The study showed that the PROACTIVE Coping Scale is a valid and reliable instrument for evaluating the proactive coping levels of nursing students.

## Introduction

The fact that nursing education programs are based on practice and that the theoretical knowledge learned in the classroom is put into practice in real clinical environments may cause nursing students to experience more stress than other university students [1, 2]. While the clinical environment offers rich opportunities for gaining practical experience, it is the greatest source of stress for nursing students [2]. The main stressors that nursing students may encounter in the clinical environment include unfamiliarity with the clinical setting, lack of confidence, fear of making mistakes, negative reactions to patients’ deaths or suffering, student-instructor relationships, and nurses’ attitudes towards nursing students. In addition to these, nursing students must cope with academic stressors related to the challenges of nursing education, exams, and evaluations [1, 2] as well as personal/social stressors related to their private lives [2–5]. Furthermore, after completing their education, nursing students face future stressors such as the transition to professional life, concerns about where and when to start their careers, and taking on financial responsibilities [6, 7].

Stress can be beneficial to individuals at minimal levels because it increases excitement and motivation. However, inadequacy in coping with stress or chronic stress can negatively affect an individual’s mental and physical health [8, 9]. Chronic stress can affect nursing students’ learning, decision-making, and thinking, ultimately impacting their academic success and even causing them to drop out of nursing education [8, 10]. Inability to manage stress and inadequacy in coping with stress can lead nursing students to experience negative conditions such as anxiety, anger, worry, tension, depression, guilt, and insomnia [9, 10]. Moreover, if adequate measures are not taken against the factors causing stress in future nurses and nursing students experience high levels of prolonged stress, it can negatively affect the workforce providing care services and the quality of patient care in the future [9]. Determining the most effective stress management methods during nursing education can increase success during education, contribute to the maintenance of knowledge and help students adapt to the nursing profession with a successful transition to professional life [7]. One of the coping strategies that nursing students can use to manage future stressors is proactive coping.

## Background

Aspinwall and Taylor (1997) defined proactive coping as “the efforts undertaken in advance to change the form of or prevent a potentially stressful event before it occurs”. Individuals who adopt proactive coping can recognize the potential difficulties around them, and they can cope with these difficulties before burnout occurs [11]. Since proactive coping behaviour is used before a potential stressor occurs, the degree of stress experienced in the case of a stressful situation can be minimized. In addition, individuals have too many coping options and sources before stressors occur; however, these options become limited after stressors occur. In addition, proactive coping includes learning from past mistakes, making plans for future goals, predicting the likelihood of stress and taking precautions [12].

Research on this topic shows that the more individuals use proactive coping strategies whenever they face stressful events, the lower their stress levels will be [13–15]. Proactive individuals are less likely to build up stress to a point where they cannot put up with it because these individuals take care to plan for the future and create resources to buffer against stress along the way. All these factors enable proactive individuals to overcome challenging goals more easily and to support their personal development [11]. The literature indicates that nurses with high proactive coping skills have a high quality of life [16], and they report less emotional burnout, less depersonalization and more personal success [11]. In a study conducted in China with nursing students, it was found that students with a highly proactive personality had a lower risk of academic burnout [17], while in another study, it was found that a proactive personality in nursing students was positively correlated with career adaptability and self-perceived employability [18].

Proactive coping is very important for nursing students to cope effectively with various stressors that they may encounter in their future education and clinical and professional lives. However, when the literature was reviewed, no valid and reliable instrument was found for measuring proactive coping levels in a sample of nurses or nursing students. Tian et al. developed the PROACTIVE Coping Scale in 2023 to measure the proactive coping levels of university students in the USA. In the analyses performed to evaluate its validity and reliability, it was determined that the scale showed strong psychometric properties [12]. This formed the starting point of the study, and the “PROACTIVE Coping Scale” was used to adapt the scale to Turkish culture, determine its validity and reliability in a sample of undergraduate nursing students, and determine the proactive coping levels of nursing students.

## Methods

### Design and sample

This research was methodologically and descriptively conducted. The universe of the research consisted of a total of 346 students studying in the second, third and fourth grades of the nursing department of a university located in the central region of Turkey. There are opinions that the number of samples in scale adaptation studies should be at least five [19], 10 [20] or 15 [21] times the total number of items. Since the number of items in the PROACTIVE Coping Scale is 19, a sample size between 95 and 285 is sufficient. However, in this study, no sample selection was made, and we planned to include the whole population. The study was completed with the participation of 272 volunteer students. The inclusion criteria for the study were having participated in clinical practice at least once and volunteering to participate in the study. Since nursing education is based on both theoretical knowledge and clinical practice, and students are exposed to numerous stress factors during their clinical practice training, only students who are currently undertaking clinical practice were included in the study. Since clinical practice started in the second year in this faculty, first-year nursing students were not included in the study.

### Instruments

The Personal Information Form and the PROACTIVE Coping Scale were used in this study as data collection instruments.

### Personal information form

This form was prepared by the researcher and included a total of 10 questions about age, gender, marital status, type of family students, year of study, state of choosing the nursing department willingly, state of loving the nursing profession, state of hesitating to take responsibility, state of coping with difficulties, and state of worrying about future stressors.

### PROACTIVE coping scale

The PROACTIVE Coping Scale was developed by Tian et al. in 2023 for university students in the USA [12]. The original version of the Proactive Coping Scale consists of four sub-dimensions and 19 items: Active Preparation (items 1, 4, 5, 7, 17, 18), which describes activities done to identify, prevent, and prepare for future stressors; Ineffective Preparation (items 3, 6, 11, 15, 19), which identifies perceived difficulties in coping with future stressors; Self-Management (items 8, 9, 12, 14), which describes regulatory activities that facilitate and sustain positive conditions while preparing for future stressors; and Utilization of Social Resources (items 2, 10, 13, 16), which questions the use of social resources to cope with future stressors. The lowest score that can be obtained from the scale is 19 and the highest score is 95. As the score obtained from the scale increases, the proactive coping levels of the participants increase. The scale is a 5-point Likert-type scale, and the participants are asked to respond to all items on a scale ranging from 1 = strongly disagree to 5 = strongly agree. In Tian et al.’s (2023) study, the Cronbach’s alpha reliability coefficients of the original scale were 0.62 for the active preparation factor, 0.81 for the ineffective preparation factor, 0.69 for the self-management factor, and 0.69 for the utilization of social resources factor. In the present study, the Cronbach’s alpha reliability coefficients were 0.816 for the total scale, 0.761 for the active preparation factor, 0.729 for the ineffective preparation factor, 0.786 for the self-management factor, and 0.658 for the utilization of social resources factor. Permission was obtained from the scale owner [12] via email to conduct validity and reliability studies on the PROACTIVE Coping Scale in the Turkish population and nursing students.

### Linguistic validity

Among the validity and reliability studies of the PROACTIVE Coping Scale, the first focused on language validity. Before language validity, permission to use the scale was obtained from Tian et al. (2023). The scale was first translated to Turkish by four translators who were fluent in English and native speakers of Turkish. Later, the scale was back-translated from Turkish to English by three translators who were fluent in both Turkish and English. The scale, which was retranslated to English, was compared with the original version, and the required revisions were made. The scale obtained after the adjustments had the same meaning as the original scale. Then, the scale was submitted to expert opinion for content validity.

### Content validity

The PROACTIVE Coping Scale was presented to ten experts (7 faculty members in the field of nursing, 1 specialist nurse, and 2 faculty members in the field of psychology) for the evaluation of content validity. In terms of content validity, the experts evaluated scale items in terms of comprehensibility, purpose, cultural appropriateness and discrimination. The Lawshe (1975) technique was used to evaluate expert opinions [22]. In the Lawshe (1975) technique, experts evaluate each scale item between points “1” and “3” [1 = Item necessary, 2 = Item useful but not sufficient, 3 = Item unnecessary). In this research, the Content Validity Ratio CVR was taken as 0.62 because of the opinions of ten experts [23]. There were no items with a CVR < 0.62 within the scope of the study. The content validity index (CVI) was calculated from the means of the CVRs and was found to be 0.95. As a result, since CVI ≥ CVR (0.95 ≥ 0.62), the content validity of the 19-item structure was statistically significant. Therefore, no item within the scope of content validity was removed.

After content validity, a pilot study was conducted with a group of 30 nursing students who were not included in the sample to evaluate the comprehensibility of the scale items. Most of the students included in the pilot study stated that the scale items were understandable. A very small number of students made suggestions regarding spelling and understandability of some items. The required revisions were made, and the scale was finalized in line with the results of the pilot study.

### Data collection and analysis

Research data were collected between 01.12.2023 and 01.01.2024 via face-to-face interviews. Before starting the research, the nursing students were informed about the research, and written and verbal consent was obtained. A total of 272 students agreed to participate in the study voluntarily. The test-retest method was used to test the invariance of the scale over time (*n* = 50). The test-retest method is the second application of the measurement tool to the sample group under the same conditions, in which individuals do not remember their responses to the scale items and there is no significant change in the characteristics to be measured [24]. This time interval is between 15 and 30 days according to the literature [25]. Therefore, the PROACTIVE Coping Scale was reapplied three weeks after the first application to 50 nursing students who were identified within the scope of the study and who were given nicknames in the first application.

In this study, the data were analysed with the SPSS 26 and AMOS 22 package programs. Reliability analyses were performed using Cronbach’s alpha coefficient, item-total score correlation and test-retest techniques. To test the validity of the scale, language validity, the content validity index and construct validity (explanatory factor analysis) were used. In addition, Kaiser‒Meyer‒Olkin (KMO) tests were used to determine the sample size, and Bartlett’s test of sphericity was used to determine the suitability of the sample volume for factor analysis. *p <* 0.05 was considered to indicate statistical significance.

### Ethical issues and permissions

Before starting the research, permission to use the scale was obtained from Tian, Tsai, Khalsa, Condie, Kopystynsky, Ohde, Zhao (2023) via e-mail. Later, ethics committee approval was received from Yozgat Bozok University Ethics Commission (Decision No: 08/11 Date: 23.11.2023) and institutional permission was obtained from the institution where the research was conducted (No: E-88148187-020-186521). In addition, before starting the application, the students were informed that the research was completely voluntary and that the content, purpose, scope and data of the research would be kept confidential. Written consent was obtained from students who voluntarily agreed to participate in the research. The study was conducted in accordance with the principles of the Declaration of Helsinki (WTO General Assembly, Fortaleza, Brazil, October 2013) and the Law on Medical Research Involving Human Subjects.

## Results

### Characteristics of the students

Among the nursing students who participated in the study (*n* = 272), 81.6% were female, 98.2% were single, 80.9% grew up in a nuclear family, 58.1% were in their third year, 58.1% chose the nursing department willingly, 67.6% loved the nursing profession, 82% were not willing to take responsibility, 91.2% believed that they could overcome difficulties and 87.5% were concerned about future stressors.

### Results of validity

#### Explanatory factor analysis

Before determining the factor structure of the PROACTIVE Coping Scale, the KMO test was used to determine whether the data were suitable for factor analysis, and the Bartlett sphericity test was used to evaluate whether the correlations between the analysed variables were significant (Table 1).

**Table 1:** Exploratory factor analysis results of PROACTIVE coping scale

Factor	Item Number	Factor Loads				Total Correlation	Explained Variance %	
		1	2	3	4			
**Active Preparation**	1	0.623				0.650	15.39	
	4	0.622				0.464		
	5	0.589				0.553		
	7	0.735				0.446		
	17	0.663				0.364		
	18	0.497				0.421		
**Ineffective Preparation**	3			0.745		0.264	13.28	
	6			0.653		0.266		
	11			0.666		0.271		
	15			0.659		0.272		
	19			0.704		0.263		
**Self-management**	8		0.758			0.442	14.31	
	9		0.809			0.321		
	12		0.648			0.473		
	14		0.701			0.415		
**Utilization of Social Resources**	2				0.605	0.485	16.61	
	13				0.522	0.530		
	10				0.716	0.436		
	16				0.704	0.318		
**Scale**							67.17	

*KMO =* 0.836 *DF = 171 χ*^*2*^ *=* 1535.000 *p <* 0.001

The KMO value of the PROACTIVE Coping Scale was 0.836 (Table 1). The KMO value is between 0 and 1 and shows a more reliable factor structure as it approaches 1. A value > 0.50 is acceptable, a value between 0.50 and 0.70 is normal, a value between 0.70 and 0.80 is good, a value between 0.80 and 0.90 is very good, and a value > 0.90 is interpreted as an excellent sample size [26]. Thus, factor analysis results are obtained with a useful and usable sample. The sample size of the scale with a KMO value of 0.836 was sufficient, and the Bartlett sphericity test results showed that the data were suitable for factor analysis (χ2 = 1535.000, *p* < 0.001) (Table 1).

Table 1 shows in detail the factor structure of the PROACTIVE Coping Scale, in which items are included in the factors and the factor loading of each item. According to these results, the factor loadings of the items vary between 0.497 and 0.735 for the active preparation factor, between 0.653 and 0.745 for the ineffective preparation factor, between 0.648 and 0.809 for the self-management factor and between 0.522 and 0.716 for the utilization of social resources factor. The table shows that the factor loadings of all the items are above 0.500 [26]. Therefore, the four factors of the proactive coping scale measure the subfeatures (Table 1).

Total correlations above 0.20 show that the item is important for the question. According to the results, the total correlation values are between 0.263 and 0.650. The results show that the survey is a valid and reliable instrument (Table 1) [27].

The explained variance ratio shows the strength of the factor structure of the scale. While the 6-item ***active preparation*** factor explained 15.39% of the total structure, the 5-item ***ineffective preparation*** factor explained 13.28% of the total structure, the 4-item ***self-management*** factor explained 14.31% of the total structure, and the 4-item ***utilization of social resources*** factor explained 16.61% of the total structure. These 4 factors and 19 items explained 67.17% of the total variance (Table 1).

#### Confirmatory factor analysis

CFA examines validity by testing the structures determined with EFA or confirming the results of a previously conducted scale with new data sets. To confirm the 19-item and 4-factor structure established as a result of exploratory factor analysis, the measurement model was analysed with CFA. The χ2/df, RMSEA, IFI, TLI, CFI and GFI were used to evaluate the factor validity of the models within the scope of CFA (Table 2).

**Table 2:** Fit index values of the measurement model of PROACTIVE coping scale

Scale	(χ^2^/sd)	RMSEA	IFI	TLI	CFI	GFI
**Model**	1.947	0.059	0.903	0.905	0.901	0.900

χ²/sd: Chi-square statistic, GFI: Goodness of fit index, IFI: Increment fit index, TLI: Trucker-Lewis Index, CFI: Comparative fit index, RMSEA: Root Mean Square Error of Approximation, SRMR: Square root of the standardised mean square error

The RMSEA is an index that is least affected by sample size. However, the RMSEA fit index criteria are inconsistent in different studies. It is thought that a cut-off value close to 0.06 or 0.08 is usually acceptable in this field. IFI, TLI, CFI and GFI fit indices exceeding 0.90 are accepted as proof of sufficient model fit [25]. In this study, the RMSEA was ≤ 0.05; the IFI, TLI, and CFI were ≥ 0.90; and the GFI was ≥ 0.85 and acceptable. The model obtained for the PROACTIVE Coping Scale (χ2/sd *=* 1.947 *dF =* 147) has four factors. The fit indices of this model show that the model has an acceptable level of fit (Table 2).

**Fig. 1:**
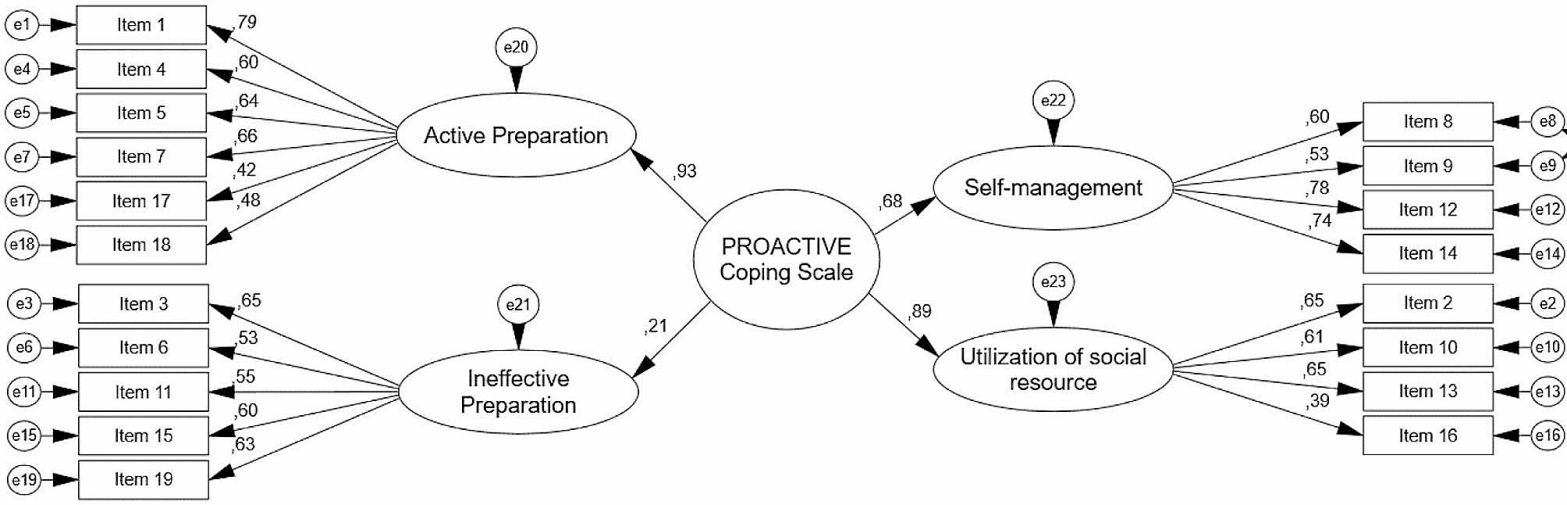
PROACTIVE Coping Scale confirmatory factor analysis model

A path diagram of the factor loading values of the confirmed measurement model is shown in Fig. 1. When the 19-item and 4-factor measurement models confirmed in this study are examined, which items make up the model and the standardized regression coefficients of the paths on the one-way arrows can be seen (Fig. 1).

Each of the path coefficients for the 19 questions was statistically significant (*p <* 0.05). According to these results, the active preparation factor consisted of questions 1, 4, 5, 7, 17 and 18; the ineffective preparation factor consisted of questions 3, 6, 11, 15 and 19; the self-management factor consisted of questions 8, 9, 12 and 14; and the utilization of social resources factor consisted of questions 2, 10, 13 and 16. All the factors had highly statistically significant effects on the questions. The path coefficients of the factors active preparation, ineffective preparation, self-management and utilization of social resources are statistically significant (*p <* 0.05). The factor with the greatest effect is active preparation, while the factor with the least effect is ineffective preparation.

In addition, item 1 is the strongest indicator of the active preparation factor, with a value of 0.79; item 3 is the strongest indicator of the ineffective preparation factor, with a value of 0.65; item 12 is the strongest indicator of the self-management factor, with a value of 0.78; and items 2 and 10 are the strongest indicators of the utilization of social resources factor, with a value of 0.65.

### Results of reliability

#### Internal consistency (Cronbach’s alpha) coefficients

The internal consistency of the PROACTIVE Coping Scale and factors were evaluated with the Cronbach’s alpha reliability coefficient. According to these results, the 19-item PROACTIVE Coping Scale is highly reliable (α = 0.816), while the factors active preparation (α = 0.761), ineffective preparation (α = 0.729) and self-management (α = 0.786) are reliable at a generally accepted level, and the factor utilization of social resources is moderately reliable (α = 0.658). The results showed that the PROACTIVE Coping Scale is a valid and reliable instrument that can be used to measure the proactive coping behaviours of nursing students [24].

#### Test-retest reliability

To determine the invariance of the scale over time, the scale was reapplied to the participants three weeks after the first application (Table 3). The correlation between the scores obtained from the first and second administration of the scale was examined with the intraclass correlation coefficient (ICC), and the results are shown in Table 3.

**Table 3:** Intraclass correlation coefficients and significance between PROACTIVE coping scale test-retest scores

		ICC	*p*
**PROACTIVE Coping Scale**		**0.817**	0.000*
**Active preparation**		0.805	0.000*
**Ineffective preparation**		0.737	0.000*
**Self-management**		0.724	0.000*
**Utilization of social resources**		0.796	0.000*

*: *p* < 0.001 ICC = intraclass correlation coefficients p = level of significance

Table 3 shows that the agreement between the responses to the questions was very good in the retest with the nursing students (*p* < 0.001). [24].

The mean score of the PROACTIVE Coping Scale was 66.02 ± 8.19, the mean score of the active preparation factor was 21.93 ± 3.41, the mean score of the ineffective preparation factor was 15.22 ± 3.383, the mean score of the self-management factor was 14.16 ± 2.83, and the mean score of the utilization of social resources factor was 14.71 ± 2.39.

The sub-dimensions of the PROACTIVE coping scale and the total score of the scale were obtained by the sum of the number of questions, and there are no reverse items in the scale. When the minimum possible score of the scale is 19, the maximum possible score is 95. The scores that the Active Preparation sub-dimension can receive vary between 6 and 30, the Ineffective Preparation sub-dimension can receive scores between 5 and 25, and the scores that the Self-Management and Social Resource Use sub-dimensions can receive vary between 4 and 20.

## Discussion

While proactive coping contributes to the personality development of nursing students, it also prepares them for the nursing profession. For this reason, it is important to popularize proactive coping in the nursing literature and to introduce relevant measurement tools. The aim of this study was to examine the validity and reliability of the “PROACTIVE Coping Scale” in nursing students, and for this purpose, language validity, content validity, construct validity, reliability analyses and internal consistency analyses were conducted.

In the scale adaptation process, the psycholinguistic properties of the scale are examined first. For linguistic validity, items of the scale should be translated first. The translation-backtranslation method is generally used for translation. To minimize the difference between the cultures in which the scale has been developed and in which adaptation is being made, translations by at least two translators who know both culture and language well are recommended in the literature [25, 28]. The translation-backtranslation method was used in this study for linguistic validity. After the required revisions were made, content validity studies were started.

Content validity is the ability of the scale items to adequately measure behaviours [29]. Expert opinions are taken for content validity. In line with the opinions of the experts, the content validity rate of the whole item varied between 0.80 and 1.00, and the content validity of the scale was calculated as 0.95. No item was removed from the scale since the difference between expert opinions was not statistically significant (*p* > 0.05).

Following linguistic and content validity studies, a pilot study is required to evaluate the comprehensibility of scale items. Pilot studies should include individuals who have the same characteristics as the sample [25]. It is recommended that the pilot study be carried out with 10% of the main sample or 30 people [24]. Accordingly, final edits were made to the items by taking the opinions of 30 nursing students who were not included in the study.

In exploratory factor analysis, the KMO test was conducted to evaluate the appropriateness of the sample size for factor analysis, and the KMO value was found to be 0.836, which shows that the sample size was “good enough” for factor analysis [30]. According to the Bartlett test results, the chi-square value was found to be acceptable and appropriate for factor analysis (χ2 = 1535.000 *p* < 0.001). As a result of the factor analysis, the factor loadings of the 4-factor PROACTIVE Coping Scale were found to be between 0.522 and 0.809. According to this result, the significance of all questions in the factor is sufficient.

CFA is used to confirm the factor structure of a scale with a predetermined factor structure [31]. In the confirmatory factor analysis, χ2/df, GFI, IFI, TLI, CFI and RMSEA were used to evaluate the model fit of the scale [29]. When the fit indices of this model were examined (χ²/sd: 1.947; GFI: 0.900; IFI: 0.903; TLI(NNFI): 0.905, CFI: 0.901; RMSEA: 0.059), it was found that the model had an acceptable fit. Therefore, 19-item and four-factor versions of the PROACTIVE Coping Scale were introduced to Turkish society.

In addition to being valid, an instrument should also be reliable. Reliability is defined as the degree to which an instrument consistently measures the phenomenon it wants to measure [29]. The Cronbach’s alpha reliability coefficient should be calculated to determine whether a Likert-type scale is reliable [32]. A Cronbach’s alpha coefficient close to 1 means that the scale is reliable [29]. In the present study, the Cronbach’s alpha reliability coefficients were 0.816 for the PROACTIVE Coping Scale, 0.761 for the active preparation factor, α = 0.729 for the ineffective preparation factor, α = 0.786 for the self-management factor and α = 0.658 for the utilization of social resources factor. According to these results, the PROACTIVE Coping Scale is a valid and reliable instrument that can be used to measure the proactive coping levels of nursing students.

Intraclass correlation coefficient values between the test-retest scores obtained to test the invariance of the PROACTIVE Coping Scale and the factors of active preparation, ineffective preparation, self-management and the utilization of social resources over time were found to be 0.817, 0.805, 0.737, 0.724 and 0.796, respectively. It has been reported in the literature that an ICC > 0.740 means that the scale has very good reliability [33]. Therefore, the PROACTIVE Coping Scale and its factors are reliable.

In the present study, the mean score of the “PROACTIVE Coping Scale” was 66.02 ± 8.19, the mean score of the “Active Preparation” factor was 21.93 ± 3.41, the mean score of the “Ineffective Preparation” factor was 15.22 ± 3.383, the mean score of the “Self-management” factor was 14.16 ± 2.83, and the mean score of the “Utilization of Social Resources” factor was 14.71 ± 2.39. Considering that the maximum possible score of the PROACTIVE Coping Scale is 95, it can be said that nursing students have above-average proactive coping levels. Similar to the results of the present study, in another study conducted in the literature, the proactive coping levels of nurses were found to be above average, and it was emphasized that nurses with high proactive coping levels had good internal control, active coping, and a high sense of personal success and self-efficacy [16]. In addition, when the literature was reviewed, it was found that highly proactive coping behaviors in nurses were correlated with low burnout [16, 34].

### Limitations

Before starting the research, the nursing students were asked what proactive coping was. However, the majority of students stated that they did not know this word. Accordingly, before the students completed the questionnaires, proactive coping was explained to the students by the researcher over a 20-minute period. The most important limitations of the research are that nursing is a new concept that students have heard of and that the research took place in the nursing department of a single university.

## Conclusion

In conclusion, the PROACTIVE Coping Scale consists of 19 items and 4 factors: active preparation (6 items), ineffective preparation (5 items), self-management (4 items) and utilization of social resources (4 items). There are no reverse-coded items in the scale. The minimum and maximum possible scores of the scale are 19 and 95, respectively. As the score of the scale increased, the proactive coping levels of the nursing students also increased. The Cronbach’s alpha of the PROACTIVE Coping Scale was 0.816. The scale explained 67.17% of the total variance.

## Data Availability

The datasets used and/or analysed during the current study are available from the corresponding author on reasonable request.
